# Effect of acupuncture and instruction on physiological recovery from maximal exercise: a balanced-placebo controlled trial

**DOI:** 10.1186/s12906-016-1213-y

**Published:** 2016-07-18

**Authors:** Paola Urroz, Ben Colagiuri, Caroline A. Smith, Alan Yeung, Birinder S. Cheema

**Affiliations:** School of Science and Health, Western Sydney University, Locked Bag 1797, Penrith, NSW 2751 Australia; School of Psychology, University of Sydney, Darlington, NSW 2006 Australia; The National Institute of Complementary Medicine, hosted by Western Sydney University, Penrith, NSW 2751 Australia

**Keywords:** Complementary medicine, Alternative medicine, Sport, Performance, Training, Ergogenic, VO_2_max

## Abstract

**Background:**

This study aimed to investigate the effect of acupuncture administered immediately following a graded exercise test (GXT) on physiological measures of recovery and determine if instruction (expectancy) affected the responses.

**Methods:**

A balanced-placebo 2 × 2 factorial design was used with treatment (real vs placebo acupuncture) and instruction (told real vs told placebo acupuncture) as factors; a no-treatment control group was also included to compare the treatment responses to no treatment. Recreationally active, acupuncture naïve young adults (*n* = 60) performed a GXT to exhaustion on a cycle ergometer (15 W/min). Heart rate, blood pressure, oxygen consumption, respiratory rate and blood lactate were collected during the test and during 60 min of supine recovery on a plinth. An experienced acupuncturist delivered real or placebo acupuncture within 6 min of completing the GXT (total treatment time = 20 min). Real acupuncture points included Neiguan (PC6), Zusanli (ST36), Lieque (LU7), and Tanzhang (REN17), while placebo acupuncture was delivered using the Park sham needle placed 1–2 cm away from each real acupuncture point. The control group received no intervention.

**Results:**

Linear and quadratic trend analyses over time indicated no significant differences between groups on any dependent variable. However, analysis of specific timepoints (every 10 min of the 60 min recovery) revealed that participants who received some form of treatment had a lower heart rate than participants in the no treatment control group (*p* = 0.042) at 20 min post-exercise. Further, a significant treatment by instruction interaction effect for heart rate was also found at 50 min (*p* = 0.042) and 60 min (*p* = 0.013) post-exercise, indicating that the differences between real and placebo acupuncture were affected by expectancy manipulation. No other significant effects were noted. However, it was interesting to note that participants who believed they were given real acupuncture reported quicker perceived recovery independent of actual treatment (*p* = 0.006) suggesting that instruction about treatment influenced perceived recovery.

**Conclusion:**

In summary, due to limited evidence, the current study does not support the acute use of acupuncture for exercise recovery. However, importantly, the current study demonstrates that a balanced-placebo design is viable for testing acupuncture and expectancy effects, and this methodology could therefore be implemented in future studies.

**Trial registration:**

ACTRN12612001015831 (Date registered: 20/09/2012).

## Background

According to a review by the World Health Organization, acupuncture may be beneficial for acute and chronic treatment of more than 40 medical conditions, including cardiovascular diseases [1]. For example, Richter *et al.* [2] have shown that acupuncture prescribed twice per week for 4 weeks can significantly reduce the frequency of angina attacks and increase the ischemic threshold in patients with angina pectoris. In patients with hypertension, a single session of acupuncture has been shown to acutely reduce resting systolic and diastolic blood pressure [3]. Studies evaluating cardiovascular responses to acupuncture have utilized many different acupoints, with *Neiguan* (PC6) and *Zusanli* (ST36) being consistently applied [3–6].

The physiologic mechanisms that underlie the cardiovascular benefits of acupuncture have not been elucidated. A shift in the cardiac autonomic balance toward greater parasympathetic (vagal) activity [7–10] and related endocrine and vascular adaptations (e.g. vasodilation) have been hypothesized as contributing factors [11]. These inferences have led to speculation that acupuncture could be applied to alter physiologic responses to exercise [12].

We recently published a systematic review suggesting that acupuncture might enhance measures of exercise performance and post-exercise recovery [12]. However, we also indicated that our data must be interpreted with caution given that we identified only four trials containing many methodological limitations. All four trials suffered from small sample size (*n* = 10 to *n* = 30), poor reporting of pertinent participant characteristics (e.g. inclusion criteria), and poor or no reporting of adverse events. Moreover, three of the studies involved a within-subjects (cross-over) design. This is a substantial limitation as the potential for failed blinding is much greater in cross-over trials where participants receive all treatments [13]. Further, the only parallel group randomized controlled trial (RCT) to date [14] may have enrolled participants who had previously experienced acupuncture; it may be more difficult to blind such participants to a placebo acupuncture condition [15].

In addition to this, a general limitation is that RCTs investigating acupuncture often find equivalent responses to real and placebo acupuncture, but that both real and placebo acupuncture are superior to no treatment. Responses observed to placebo conditions may result from stimulation induced by the sham acupuncture and/or participant expectancy, which can produce improvement *via* the placebo effect [15]. Indeed, there is some research that suggests that the expectancy of the patient is a fundamental factor contributing to the effectiveness of acupuncture [16, 17]. However, few studies involving acupuncture have directly examined the relationship between expectancies and treatment responses in a balanced design in which both these factors are manipulated [15]. Therefore, the relative contribution of each factor remains unclear.

The present study aimed to investigate the effect of acute (20 min) acupuncture treatment administered immediately after a maximal exercise test on physiological measures of recovery, including heart rate (HR), systolic- (SBP) and diastolic blood pressure (DBP), volume of oxygen consumption (VO_2_), respiratory rate (RR), and blood lactate (BL). This study attempted to overcome the limitations of previous research [12] by investigating an acupuncture naïve cohort and employing a balanced-placebo 2 × 2 design [18] with treatment and instruction as factors. The major advantage of this design is that it can dissociate improvements caused by acupuncture from those caused by the placebo effect, if any [15]. To our knowledge, no study to date has used a balanced placebo design to investigate the efficacy of acupuncture treatment.

## Methods

### Study design

This study employed a randomized, balanced-placebo 2 × 2 factorial design plus a no-treatment control group [18]. The first factor was acupuncture with participants allocated to receive Traditional Chinese Medicine (TCM) acupuncture or placebo acupuncture. The second factor was instruction (expectancy) with half of the participants told that they had been given real acupuncture and the other half being told they have been given placebo acupuncture, irrespective of their actual treatment. Thus, the study involved a total of five groups: (1) an acupuncture with high expectancy group (A-Hi), (2) an acupuncture with low expectancy group (A-Lo), (3) a placebo acupuncture with high expectancy group (P-Hi), (4) a placebo acupuncture with low expectancy group (P-Lo) and (5) a control (no treatment) group (C). The University of Western Sydney Human Research Ethics Committee approved all procedures and all participants provided written informed consent.

### Sample size and power calculation

Our sample size estimates were driven by the hypothesized difference in HR at 30 min of recovery from maximal exercise in the A-Hi versus P-Lo groups based on a previous randomized controlled trial [14]. The data suggest that the A-Hi group were expected to have a HR that was 2.3 beats/min lower than the P-Lo group at 30 min post (i.e. 80.0 ± 1.24 versus 82.3 ± 1.25) resulting in an effect size of 1.8. Using a two-tailed test of significance, setting the alpha set at 0.05 and beta at 0.20, approximately 85 participants (17 per group) would be required to have 80 % power to detect a large effect (ES = 1.0) between the A-Hi and P-Lo groups.

### Participants

Inclusion criteria: Adults (18–30 years); recreationally active (i.e. engaged in moderate to vigorous physical activity for a minimum of 30 min per session, three sessions per week); no previous use of acupuncture; no phobia of needles; no medication other than the contraceptive pill. Participants were recruited by means of convenience sampling *via* advertisements on the university campus and website.

### Protocol and outcome measures

All participants were instructed to abstain from alcohol and exercise for >24 h, and cigarettes and caffeine for >4 h prior to testing.

The protocol involved three phases:

#### Baseline resting condition (15 min)

Participants were instructed to lie in a supine position on a plinth for 15 min to evaluate resting parameters and establish a standardized baseline. HR was measured *via* telemetry (Polar XL, Stamford, CT) and SBP and DBP were measured *via* auscultation at 5, 10 and 15 min. VO_2_ and RR were monitored continuously using a Masterscreen CPX metabolic cart (Jaeger, Würzburg, Germany) breath-by-breath analysis protocol with the sampling frequency set at 15 s. Before each testing session the O_2_ and CO_2_ sensors were calibrated using high-grade calibration gas with certified concentrations (O_2_ = 16 %, CO_2_ = 5 % and N_2_ = balance). BL was collected from the earlobe onto a lactate strip (Cobas® BM-Lactate coded strips) and was analyzed using a Lactate Pro (Roche Diagnostics, Accutrend® Lactate) at 10 min. The same procedures applied over the next two phases.

#### Graded exercise test

Immediately after supine rest, participants were transferred to an electronically braked cycle ergometer (Velotron, Racermate, Boulder, CO, USA) to perform a GXT to volitional fatigue (exhaustion) using a ramped protocol [14, 19–21]. The cycle resistance was set to 0 Watts (W) with increments increasing 15 W/min. Participants were required to maintain a cadence of 60 revolutions per min (RPM). The GXT was terminated if the participant could no longer maintain the required cadence, or if the participant requested to terminate the test. HR and SBP/DBP were recorded every 3 min during the GXT, while VO_2_, and RR were monitored continuously. Ratings of perceived exertion [22] was also measured during the exercise test at 3 min intervals and when the test was terminated.

#### Post-exercise recovery (60 min)

Immediately after the GXT, the participant was instructed to return as quickly as possible to a supine position on the plinth; peak HR and SBP/DBP measures were then collected. The participant was then randomized to one of five groups where treatment and expectancy factors were manipulated (detailed in next section). Randomization assignments were pre-determined in blocks of ten *via* randomization program (www.randomization.com) in a concealed envelope by an investigator not involved in the testing sessions. Researchers present during the testing sessions were blinded to the arrangement of randomization. During the supine recovery phase, HR and BP were recorded every 5 min (commencing at 5 min), while VO_2_ and RR were monitored continuously. BL was measured 5 min post-exercise and every 10 min thereafter. For participants randomized to one of the four treatment conditions, the testing session was followed by a survey regarding their experience of pain, pain intensity, recovery, and benefit.

### Acupuncture treatments and instruction

Based on pilot data, the length of time from the start of recovery (assuming the supine position as quickly as possible) to full needle insertion or placebo application was 6 min. A single session of the study intervention was administered. The acupuncture needles or placebo device were retained for a total of 20 min inclusive of the time taken to insert the needles. An experienced acupuncturist registered with the Chinese Medicine Board of Australia delivered the real and placebo treatments. The acupuncturist had a bachelor’s degree in TCM from Western Sydney University that included training at the Jiangsu Provincial Hospital in China, plus two additional years of clinical experience. No other interventions were administered.

#### Real acupuncture

Based on previous literature [14, 19–21], and consultations with acupuncturists from China, TCM was identified as the most appropriate style of acupuncture for this study. Four primary acupoints were administered including *Neiguan* (PC6), *Zusanli* (ST36), *Lieque* (LU7), and *Tanzhang* (REN17). All acupoints were carried out bilaterally except REN17. The rationale for these points was based on the consistent physiological effect of PC6 and ST6 on cardiovascular function [2, 23, 24], while LU7 is applied for promoting lung function [25] and REN17 is indicated for regulating *Q*i in the chest and treating breathlessness [26]. PC6, ST36, and LU7 have been used in previous research evaluating the effect of acupuncture on exercise recovery [14, 27] and exercise performance [21]. There was no individual variation within the protocol. Single-use disposable stainless Sensei© steel needles (0.20 × 40 mm for body acupuncture) were used. Acupuncture needles were inserted to tissue level and stimulated manually after needle insertion, at 10 min, and prior to removal to achieve and maintain deqi sensations.

#### Placebo acupuncture

Placebo acupuncture was delivered using the Park sham needle [28], which has been shown to be indistinguishable from the same procedure using real needles in acupuncture naïve participants [28]. The device contains a retractable needle and blunt tip to ensure no penetration of the skin. The sham needles were placed 1–2 cm away from the real acupoints.

#### Instruction

Instruction was delivered verbally using standardized scripts. Participants randomized to receive A-Hi or P-Hi were informed that they would be receiving real acupuncture. Participants randomized to receive A-Lo or P-Lo were informed that they would be receiving placebo acupuncture. Participants randomised to the control (no treatment) group were informed that they would be receiving no treatment.

### Statistical analyses

Analyses was performed using the *Statistical Package for the Social Sciences* (IBM©, SPSS Version 20.0). Between group comparisons of pertinent demographic variables (e.g. gender, age, BMI) at baseline and maximal exercise data were analyzed using univariate analysis of variance. Demographic variables found to differ significantly were included as covariates in the main analyses. One-way ANCOVAs were used to confirm that there were no differences in physiological outcomes at Peak (fatigue) point. Treatment effects were then tested in two ways. First, trend analysis was used to compare the overall rates of recovery between groups. This involved comparing linear and quadratic trends across groups as follows: Comparison A) any treatment versus no treatment, Comparison B) real acupuncture versus placebo acupuncture, Comparison C) being told real acupuncture versus being told placebo acupuncture, and Comparison D) the interaction between type of acupuncture and instruction. Second, the above four comparisons were tested for individual timepoints while controlling for peak exercise values, allowing for determination of the exact times at which the treatment/instruction may be beneficial, if any. HR, SBP, DBP VO_2_, RR, and RER were analysed at 10 min intervals from the start of supine recovery, while BL was analysed at 15 min intervals. All analyses controlled for the peak value of that particular outcome during the exercise test. Two-way ANOVAs were used to test reported pain scores, the effect of type of acupuncture, instruction, and their interaction on subjective feelings of recovery and perceived efficacy of the treatment at the end of the recovery period. A Chi-squared test of independence was used as a manipulation check. A *p* value of <0.05 was considered indicative of statistical significance.

## Results

### Participants

**Fig. 1:**
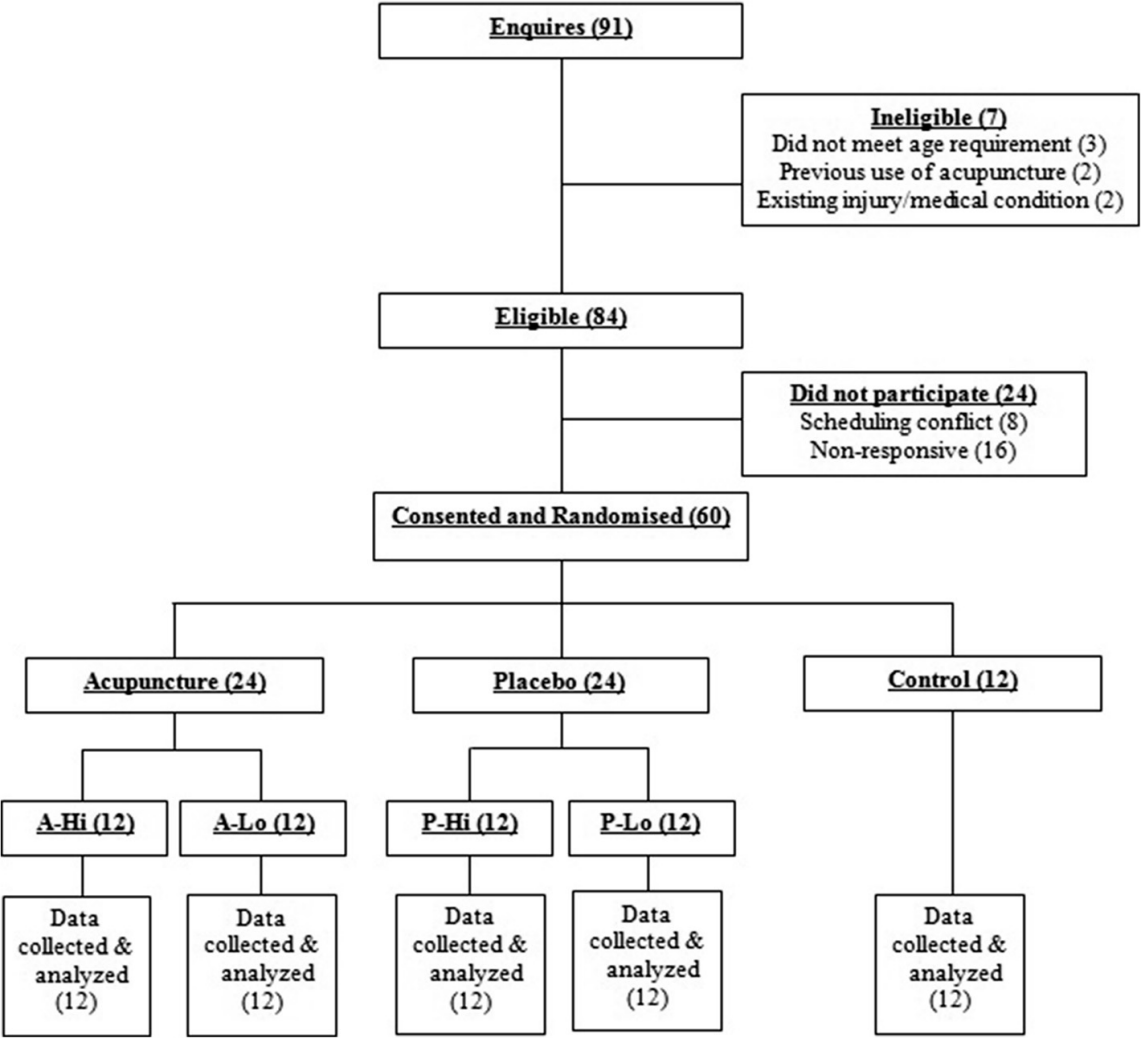
Flow of participants

Ninety-one individuals expressed interest in the study. Thirty-one were not enrolled due to ineligibility, scheduling conflicts, or being un-responsive (Fig. 1). Sixty participants participated in the study and completed all procedures. Descriptive characteristics for the total cohort and each of the five groups are presented in Table 1. The cohort ranged in age from 18 to 28 years. BMI ranged from 17.1 to 33.3 kg/m^2^. None of the participants were diagnosed with any chronic illness. All participants engaged in ≥3 moderate-intensity exercise sessions per week. All participants were naïve to acupuncture. There were no statistically significant differences between groups in the characteristics presented (Table 1).

**Table 1:** Participant characteristics

	Total Cohort	A-Hi	A-Lo	P-Hi	P-Lo	Control
Age (yrs)	21.1 ± 2.1	22 ± 2.7	20.6 ± 2.1	20.1 ± 1.8	21.3 ± 1.5	21.8 ± 1.9
Gender (M:F)	38:22	7:5	8:4	8:4	7:5	8:4
Height (cm)	175.8 ± 9.5	173.7 ± 12.8	178.8 ± 5.7	173.5 ± 6.6	175.6 ± 11.3	177.5 ± 9.6
Weight (kg)	75.2 ± 13.5	72.9 ± 12	83.9 ± 12.1	74 ± 13.2	69.3 ± 15.6	75.6 ± 11.7
BMI (kg/m^2^)	24.2 ± 3.5	24.2 ± 3.4	26.2 ± 3.3	24.5 ± 3.4	22.2 ± 2.9	24.1 ± 3.9

All data presented as mean +/− standard deviation except gender (presented as *n*)

*A-Hi* real acupuncture with high expectancy, *A-Lo* real acupuncture with low expectancy, *P-Hi* placebo acupuncture with high expectancy, *P-Lo* placebo acupuncture with low expectancy, *C* control (no treatment), *BMI* body mass index

### Physiological measures

#### Graded exercise test

All participants completed the GXT. Peak values were recorded across all variables (HR, SBP, DBP, VO_2_, RR and BL) upon termination of the test as displayed in Table 2. One-way ANOVAs comparing physiological outcomes at fatigue revealed no statistically significant differences between the (lowest *p* = 0.36).

**Table 2:** Peak exercise values at the completion of the graded exercise test

	A-Hi	A-Lo	P-Hi	P-Lo	Control
HR (bpm)	184 ± 12	178 ± 12	179 ± 12	176 ± 12	184 ± 12
SBP (mmHg)	145 ± 14	144 ± 14	144 ± 14	144 ± 13	147 ± 14
DBP (mmHg)	76 ± 6	76 ± 6	77 ± 6	78 ± 6	76 ± 6
VO_2_ (ml/min)	2393 ± 687	2577 ± 678	2393 ± 693	2558 ± 673	2356 ± 679
RR (breaths/min)	39 ± 9	38 ± 9	41 ± 9	39 ± 9	37 ± 9
BL (mmol/L)	4.55 ± 3.37	4.08 ± 3.33	3.90 ± 3.4	5.06 ± 3.3	4.51 ± 3.33

All data presented as mean +/− standard deviation

*HR* heart rate, *SBP* systolic blood pressure, *DBP* diastolic blood pressure, *VO2* volume of oxygen consumption, *RR* respiratory rate, *RER* respiratory exchange ratio, *BL* blood lactate

#### Trend analyses

Liner and quadratic trend analyses over time indicated no significant differences on any of the dependent variables for all four comparisons (Table 3).

**Table 3:** Trend analyses p-values

Comparison	HR		SBP		DBP		VO_2_		RR		BL	
	Linear	Quadratic	Linear	Quadratic	Linear	Quadratic	Linear	Quadratic	Linear	Quadratic	Linear	Quadratic
Treatment vs. No treatment	0.819	0.644	0.669	0.856	0.456	0.760	0.720	0.531	0.524	0.906	0.601	0.496
Real acupuncture vs. Placebo acupuncture	0.246	0.807	0.840	0.719	0.770	0.516	0.919	0.954	0.432	0.627	0.985	0.716
Hi- expectancy vs. Lo- expectancy	0.747	0.734	0.879	0.846	0.657	0.979	0.403	0.403	0.620	0.264	0.894	0.319
Group interaction	0.056	0.904	0.566	0.832	0.731	0.822	0.846	0.948	0.312	0.713	0.182	0.288

*HR* heart rate, *SBP* systolic blood pressure, *DBP* diastolic blood pressure, *VO2* volume of oxygen consumption, *RR* respiratory rate, *RER* respiratory exchange ratio, *BL* blood lactate

#### Timepoint analyses

Raw data tables for each of the six physiological variables are presented in Tables 4 (HR/SBP/DBP/VO_2_/RR) and 5 (BL).

**Table 4:** Physiological outcome measures

	A-Hi	A-Lo	P-Hi	P-Lo	Control
*HR (bpm):*					
Fatigue	184 ± 12	178 ± 12	179 ± 12	176 ± 12	184 ± 12
10 min	94 ± 12	95 ± 12	96 ± 12	89 ± 12	97 ± 12
20 min	88 ± 10	86 ± 10	88 ± 10	81 ± 10	94 ± 10
30 min	83 ± 11	80 ± 10	82 ± 11	78 ± 10	86 ± 10
40 min	79 ± 12	80 ± 12	81 ± 12	75 ± 12	84 ± 12
50 min	76 ± 9	75 ± 9	81 ± 9	71 ± 9	80 ± 9
60 min	72 ± 9	76 ± 9	78 ± 10	69 ± 9	79 ± 9
*SBP (mmHg):*					
Fatigue	145 ± 14	144 ± 14	144 ± 14	144 ± 13	147 ± 14
10 min	119 ± 9	121 ± 9	117 ± 9	117 ± 9	120 ± 9
20 min	113 ± 8	114 ± 8	116 ± 8	113 ± 8	116 ± 8
30 min	111 ± 9	112 ± 9	111 ± 9	112 ± 9	114 ± 9
40 min	111 ± 9	112 ± 9	112 ± 9	110 ± 9	112 ± 9
50 min	109 ± 9	111 ± 9	111 ± 9	109 ± 9	111 ± 9
60 min	109 ± 9	111 ± 9	109 ± 9	109 ± 9	110 ± 9
*DBP (mmHg):*					
Fatigue	76 ± 6	76 ± 6	77 ± 6	78 ± 6	76 ± 6
10 min	67 ± 7	71 ± 7	73 ± 7	73 ± 7	72 ± 7
20 min	70 ± 7	71 ± 7	74 ± 7	73 ± 7	72 ± 7
30 min	70 ± 7	72 ± 7	73 ± 7	75 ± 7	73 ± 7
40 min	71 ± 7	72 ± 7	72 ± 7	73 ± 7	73 ± 7
50 min	71 ± 7	72 ± 7	73 ± 7	73 ± 7	74 ± 7
60 min	70 ± 7	71 ± 7	73 ± 7	74 ± 7	73 ± 7
$\overset{.}{V}$ *O*_*2*_ *(ml · min* ^*−1*^ *)*_*:*_					
Fatigue	2393 ± 687	2577 ± 678	2393 ± 693	2558 ± 673	2356 ± 679
10 min	417 ± 114	457 ± 112	429 ± 115	402 ± 111	399 ± 112
20 min	350 ± 103	386 ± 102	351 ± 104	343 ± 101	343 ± 102
30 min	319 ± 106	387 ± 105	346 ± 107	342 ± 104	325 ± 105
40 min	334 ± 104	344 ± 103	323 ± 104	329 ± 101	330 ± 102
50 min	298 ± 106	349 ± 105	347 ± 107	305 ± 104	274 ± 105
60 min	288 ± 106	350 ± 105	325 ± 107	296 ± 104	280 ± 105
*RR (breaths/min):*					
Fatigue	39 ± 9	38 ± 9	41 ± 9	39 ± 9	37 ± 9
10 min	22 ± 27	23 ± 27	21 ± 28	23 ± 27	22 ± 27
20 min	18 ± 4	19 ± 4	19 ± 4	20 ± 4	18 ± 4
30 min	17 ± 4	19 ± 4	17 ± 4	19 ± 4	17 ± 4
40 min	17 ± 4	17 ± 4	15 ± 4	18 ± 4	16 ± 4
50 min	15 ± 4	17 ± 4	15 ± 4	18 ± 4	15 ± 4
60 min	16 ± 4	16 ± 4	16 ± 4	17 ± 4	16 ± 4
*BL (mmol/l):*					
Fatigue	4.55 ± 3.37	4.08 ± 3.33	3.90 ± 3.4	5.06 ± 3.3	4.51 ± 3.33
15 min	4.55 ± 2.81	3.17 ± 2.78	3.61 ± 2.84	4.62 ± 2.76	3.92 ± 2.78
30 min	3.08 ± 2.39	2.08 ± 2.36	2.20 ± 2.41	3.21 ± 2.34	2.33 ± 2.36
45 min	1.74 ± 2.01	1.75 ± 1.99	1.55 ± 2.03	1.82 ± 1.97	1.20 ± 1.99
60 min	0.84 ± 1.48	1.05 ± 1.46	1.06 ± 1.49	1.45 ± 1.45	0.91 ± 1.46

All data presented as mean +/− standard deviation

*HR* heart rate, *SBP* systolic blood pressure, *DBP* diastolic blood pressure, *VO2* volume of oxygen consumption, *RR* respiratory rate, *RER* respiratory exchange ratio, *BL* blood lactate

##### Heart rate

Participants who received some form of treatment had a lower HR than participants in the no treatment control group (*p* = 0.042) at 20 min post-exercise (Comparison A). Furthermore, a significant acupuncture x instruction interaction effect was found at 50 min (*p* = 0.042) and 60 min (*p* = 0.013) post-exercise, indicating that the differences between real and placebo acupuncture differed by expectancy manipulation (Comparison D). No additional significant findings or trends were evident.

##### Systolic blood pressure

No significant differences or trends were found amongst the four key comparisons across each individual post-exercise recovery timepoint controlling for baseline, indicating that treatment and/or expectancy factors did not cause any differences in SBP.

##### Diastolic blood pressure

No significant differences were found amongst the four key comparisons across each individual post-exercise recovery timepoint controlling for baseline. However, a trend was noted when comparing real acupuncture and placebo acupuncture; participants receiving real acupuncture had lower DBP compared with those receiving placebo acupuncture regardless of expectancy at 10 min (*p* = 0.058) and 30 min (*p* = 0.097) post-exercise (Comparison B).

##### Volume of oxygen consumption (VO_2_)

No significant differences or trends were found amongst the four key comparisons across each individual post-exercise recovery timepoint controlling for baseline, indicating that treatment and/or expectancy factors did not cause any differences in VO_2_.

##### Respiratory rate

No significant differences were found amongst the four key comparisons across each individual post-exercise recovery timepoint controlling for baseline. Although, after 50 min of recovery a trend (*p* = 0.052) showing that those who were manipulated to have high expectancy had lower RR than those manipulated to have low expectancy appeared, independently of the type of acupuncture (Comparison C).

##### Blood lactate

No significant differences or trends were found amongst the four key comparisons across each individual post-exercise recovery timepoint controlling for baseline, indicating that treatment and/or expectancy factors did not cause any differences in BL.

### Survey outcomes

#### Pain

Twenty three percent (*n* = 11/48) reported experiencing some degree of pain (seven acupuncture vs four placebo). Intensity of pain, if reported, ranged from 1/10 (*n* = 4) to 7/10 (*n* = 1). There were no statistically significant differences between the four treatment groups in the reporting of pain or pain intensity.

#### Manipulation check

Of the 48 participants that were randomly allocated to receive either real acupuncture or placebo acupuncture 47 participants (98 %) chose the type of treatment they were instructed they would be receiving, irrespective of the actual treatment they received. Only, one participant who received placebo acupuncture with low expectancy reported receiving real acupuncture. There were no differences in patterns of beliefs about treatment for real versus placebo acupuncture (*p* = 0.77).

#### Perceived recovery and benefit of acupuncture

Participants reported a high level of feeling recovered (mean: 9/10) and this did not differ across the treatment groups. However, when analyzing perceived efficacy of their treatment, those who were instructed to receive real acupuncture (high expectancy) reported that treatment helped them to recover quicker than normal compared to those instructed to receive placebo acupuncture (low expectancy) (*p* = 0.006).

## Discussion

This is the first known balanced-placebo controlled trial to investigate the effects of acupuncture and instruction applied following a maximal exercise test on physiological measures of recovery. Both linear and quadratic analyses indicated no significant main effects of treatment overall, acupuncture, or instruction, nor any acupuncture by instruction interaction on any of the physiological outcome measures. However, time point analysis revealed a handful of statistically significant findings and trends amongst particular outcomes at specific time points. These were a) significantly lower HR 20 min post-exercise for all treatments on average relative to the no treatment, b) a main effect trend for lower DBP following real versus placebo acupuncture at 10 min (*p* = .058) and 30 min (*p* = 0.097), and c) a main effect trend for those in the high expectancy group to have lower RR values at 50 min post-exercise than low expectancy (*p* = 0.052). This suggests limited effect of acupuncture and instruction on exercise recovery. However, the study has a number of important methodological and practical implications for future research in this area.

To our knowledge, there is only one other study that has investigated the effects of acupuncture on recovery from maximal exercise. Lin *et al.* [14] recruited and randomized 30 elite male basketball players to three groups: real acupuncture, placebo acupuncture, and a no treatment control group. The real and placebo acupuncture were applied for 15 min *prior* to the maximal exercise test, with real acupuncture applied at two points (PC6 and ST36) and placebo acupuncture involving needles inserted 1 cm away from these two points. They found that real acupuncture significantly reduced HR, VO_2,_ at 30 min and BL at 30 min and 60 min relative to placebo acupuncture and the control group.

There are a number of differences in methodology that may explain the discordant results between their study and the current study. First, we applied treatment post-exercise, rather than before. This was an intentional decision in order to attempt to isolate acupuncture and instruction effects on exercise recovery only, rather than exercise capacity. However, one practical limitation to this was that application of the treatment in our study took approximately 6 min, during which time a large amount of recovery has already occurred, e.g. a decrease in HR of 15–20 bpm towards resting. As such, we may have missed a critical phase in the recovery cycle. In addition to the two points used by Lin et al. [14], we included stimulation at LU7 or REN17 points, based on other research implicating these points in cardiovascular function [21, 26, 29]. We consider inclusion of these additional points a strength of our study, nonetheless, we cannot rule out that including them influenced the overall treatment response. The current study required participants to be acupuncture naïve whereas Lin et al. [14] did not specify their participants’ level of prior experience with acupuncture. Finally, their study involved elite athletes whereas our study involved those who engage in regular exercise but who are not necessarily elite athletes and this could translate into differences in treatment responsiveness.

Putting aside these methodological differences, the overall pattern from both Lin et al’s [14] and the current study suggests fairly inconsistent effects of acupuncture on exercise recovery, with only some outcomes being affected and only at some very specific timepoints. This could indicate two possibilities. First, given the number of outcomes and timepoints included in both studies, the statistically significant effects could simply reflect Type 1 errors due to multiple comparisons. On the other hand, it could be the case that both studies were underpowered to detect more genuine effects of acupuncture (or instruction). We powered our study based on Lin *et al’s* [14] HR data, but it could be the case that other variables are less sensitive. As such, we recommend that future studies investigating acupuncture for exercise recovery use substantially larger sample sizes. Further to reduce the possibility of Type I errors these studies could power themselves based on including control for multiple comparisons.

The inclusion of instruction as a factor in our study was a major methodological advance over previous studies. This is because the placebo effect has been suggested to account for some of acupuncture’s effects, based on findings that real and placebo acupuncture often produce equivalent outcomes that are both superior to no treatment [15, 30]. For example, the current study employed the park device [28], which involves a blunt retractable needle placed in a holding device, as the placebo acupuncture method. Claiming this as being ‘physiologically inert’ cannot be guaranteed, as literature suggest that a simple touch of the skin can cause a physiological reaction in the body [15, 30]. In the current study we found very little evidence that expectancy influenced physiological indices of exercise recovery. There are three possibilities that may explain this. First, it could be the case that the instructions did not induce sufficient expectancies to produce an effect. We intentionally chose not to assess expectancies, as we did not want to make participants suspicious about the instructions. However, this means we cannot be sure of whether instructing participants that they were receiving real acupuncture caused them to expect improvement. Second, even if the instruction manipulation was successful at influencing expectancies, as with the acupuncture effect, the study may have lacked sufficient power to detect a genuine effect of expectancy on exercise recovery. Finally, it may simply be that expectancy does not affect exercise recovery. However, given a fairly large evidence base indicating placebo effects for many health conditions, including cardiovascular function (see [31] for a review) the latter may seem less likely. To this end, it was interesting to note that participants told that they were receiving real acupuncture reported that they felt their treatment was more effective than those told that they were given placebo acupuncture as shown in previous studies [32]. This could indicate that expectancy did influence some subjective aspect of recovery, not captured by the physiological measures. Of course, on the other hand, it could indicate that instructions lead to bias in subjective reports of recovery and other outcomes if participants told they were being given a real treatment felt pressure to report improvement even in the absence of any, labelled demand characteristics (see [33] for a review). Whichever is the case, the current study demonstrates a viable and important method for attempting to disentangle the effects of acupuncture from expectancy in future studies.

## Conclusion

In summary, we found limited evidence that acupuncture or instruction can improve physiological measures of recovery from maximal exercise. As such, the current study does not support the acute use of acupuncture for exercise recovery. However, a number of key methodological considerations emerged that should be addressed in future research. These included the timing of the intervention (i.e. before or after exercise), which acupoints to stimulate, the characteristics of the sample (i.e. elite athletes versus regular exercisers), and power considerations. Importantly, the current study demonstrates that a balanced-placebo design is viable for testing acupuncture and expectancy effects, and this methodology could therefore be implemented in future studies.

## Abbreviations

A-Hi, acupuncture with high expectancy group; A-Lo, acupuncture with low expectancy group; ANCOVA, analysis of covariance; ANOVA, analysis of variance; BL, blood lactate; C, control (no treatment) group; DBP, diastolic blood pressure; ES, effect size; GXT, graded exercise test; HR, heart rate; LU7, Lieque; PC6, Neiguan; P-Hi, placebo with high expectancy group; P-Lo, placebo acupuncture with low expectancy group; RCT, randomized controlled trial; REN17, Tanzhang; RPM, revolutions per minute; RR, respiratory rate; SBP, systolic blood pressure; ST36, Zusanli; TCM, Traditional Chinese Medicine; VO2, volume of oxygen consumption; W, watts
